# Changes in histopathology and cytokeratin AE1/AE3 expression in skin graft with different time on Indonesian local cats

**DOI:** 10.14202/vetworld.2017.662-666

**Published:** 2017-06-18

**Authors:** Erwin, Etriwati, Gunanti, Ekowati Handharyani, Deni Noviana

**Affiliations:** 1 Laboratory of Clinic and Surgery, Faculty of Veterinary Medicine, Syiah Kuala University, Banda Aceh, Indonesia; 2 Laboratory of Pathology, Faculty of Veterinary Medicine, Syiah Kuala University, Banda Aceh, Indonesia; 3 Department of Clinic Reproduction and Pathology, Faculty of Veterinary Medicine, Bogor Agricultural University, Bogor, Indonesia

**Keywords:** cats, cytokeratin AE1/AE3, histopathology, skin graft

## Abstract

**Aim:**

A good skin graft histopathology is followed by formation of hair follicle, sweat gland, sebaceous gland, blood vessel, lightly dense connective tissue, epidermis, and dermis layer. This research aimed to observe histopathology feature and cytokeratin AE1/AE3 expression on cat skin post skin grafting within a different period of time.

**Materials and Methods:**

Nine male Indonesian local cats aged 1-2 years old weighing 3-4 kg were separated into three groups. First surgery created defect wound of 2 cm × 2 cm in size to whole groups. The wounds were left alone for several days, differing in interval between each group, respectively: Group I (for 2 days), Group II (for 4 days), and Group III (for 6 days). The second surgery was done to each group which harvested skin of thoracic area and applied it on recipient wound bed. On day 24^th^ post skin graft was an examination of histopathology and cytokeratin AE1/AE3 immunohistochemistry.

**Results:**

Group I donor skin’s epidermis layer had not formed completely whereas epidermis of donor skin of Groups II and III had completely formed. In all group hair follicle, sweat gland, sebaceous gland, and neovascularization were found. The density of connective tissue in Group I was very solid than other groups. Cytokeratin AE1/AE3 expression was found on donor skin’s epithelial cell in epidermis and dermis layer with very brown intensity for Group II, brown intensity for Group II, and lightly brown for Group I.

**Conclusion:**

Histopathological structure and cytokeratin AE1/AE3 expression post skin graft are better in Groups II and III compared to Group I.

## Introduction

Skin transplant is a transfer of skin tissue from healthy body part to be placed on wounded site. Physiological recovery of skin transplant goes through imbibition phase, revascularization phase, and maturation phase. Histopathology of wound recovery begins from cell regeneration until organ function return. Cells jointly interact, work, and function normally. Donor skin for skin graft has no blood vessel connection with wound bed. This blood vessel at the base of the wound must heal rapidly and develop so that donor skin can fuse with recipient skin. If blood vessels do not grow and develop well, donor skin will undergo necrosis [1]. Autograft and isograft can easily be conducted to animal [2]. Allograft and xenograft method of skin transplant have often been specifically described in small animal [3]. Based on subjective and objective clinical observation result of auto-skin graft in Indonesian local cats, donor skin is well received by recipient base wound [1].

Wound healing process includes hemostasis, inflammation, migration, proliferation, and remodeling [4]. Skin transplant recovery involves vascular and cellular response. Vascular response begins from the fusion of donor skin with base wound by fibrin clotting. Fibrin clotting is biological glue, which connects donor skin and base wound. Leukocytes move into fibrin clot and destroy foreign object, then exudative reaction coupled by granulation tissue growth happen. Granulation will then be followed by re-epithelialization. Re-epithelialization is described as keratinocyte reconstruction in forming epidermis layer to cover wound and return skin function. Epidermis layer is shaped by five layers of squamous epithelial cell, in which the most common among the cells is keratinocyte. The formation of epidermis and dermis layer is also followed by the development of other cell organelles. Keratinocyte is cells responsible for the formation of keratin structural protein of skin, hair, and nail [5].

Wound recovery is a complex process not exclusive from cytokine and growth factor’s influence. The role of cytokine in cellular response includes inflammation, cell proliferation, angiogenesis, extracellular matrix synthesis, and cell degradation in repairing the balance between negative and positive signal from saturated protein mediator [6]. Cytokeratin has an important role in characterizing cell differentiation [7]. Cytokeratin is a protein with an intermediate filament made of keratin, which can be found in intracytoplasmic cytoskeleton of epithelial tissue. Cytokeratin expresses epithelial cell type depending on the site it is given [5,8]. Cytokeratin AE1/AE3 is a mix of two different clones of cytokeratin monoclonal antibody. Cytokeratin AE1 detects high molecular weight cytokeratin 10, 14, 15, and 16 and also low molecular weight cytokeratin 19. Cytokeratin AE3 detects high molecular weight cytokeratin 1, 2, 3, 4, 5, and 6 and also low molecular weight cytokeratin 7 and 8 [9]. The earliest stage of wound recovery is keratinocyte migration to the edge of the wound through gaps in the wound [10]. Because of that reason, keratinocyte migration is the basic of wound recovery, which then followed by fibroblast proliferation and granulation tissue formation [8]. The desired histopathological feature from skin graft recovery is the perfect formation of epidermis and dermis tissue, hair follicle, sweat gland, sebaceous gland, blood vessel, and low-density connective tissue [1].

This research aims to observe donor skin tissue’s morphology structure with hematoxylin-eosin (HE), connective tissue formation by Masson trichrome (MT) staining, and cytokeratin AE1/AE3 expression on Indonesian local cats with a different period of donor skin placement.

## Materials and Methods

### Ethical approval

This research has been approved by the Animal Care and Use Committee of Veterinary Teaching Hospital, Faculty of Veterinary Medicine, Bogor Agricultural University (IPB) with approval number: 19-2014 IPB.

### Research procedure

Nine clinically sound cats were acclimated in individual cages for 1 month. They were fed 3 times a day with water given *ad libitum*. As acclimatization drugs, 1 month before surgery the cats were given 15 mg/kg body weight (BW) of amoxicillin and 3 mg/kg BW of clavulanic acid antibiotics (Claneksi^®^, Sanbe Farma, Bandung, Indonesia), metronidazole (Flagyl^®^, Boehringer Ingelheim Indonesia, Bogor, Indonesia) 17 mg/kg BW, praziquantel and pyrantel embonate (Drontal^®^, Bayer, USA) 5 mg/kg BW, and vitamin supplements. The cats were bathed and fasted for 8 h before surgery for vomiting prevention. Premedication given was atropine sulfate 0.25% (Atropine^®^, Ethica, Indonesia) 0.04 mg/kg BW. As general anesthetic, the cats were given combination of ketamine 10% (Ketamil^®^, Troy Laboratories PTY Limited, Australia) 10 mg/kg BW and xylazine 2% (Xyla^®^, Interchemie, Holland) 1 mg/kg BW [11].

Before surgery, hair shaving and disinfection of lateral area of the forelimb were done. Afterward, an incision wound defect 2 cm × 2 cm was made. The wound was wrapped by sterile gauze with povidone iodine then left alone with duration differing between treatment groups; Group I for 2 days, Group II for 4 days, and Group III for 6 days. Second surgery was done to harvest skin by the thoracic area and apply it on the base of recipient bed, which was cleaned of subcutaneous tissue. The donor skin and recipient skin were then stitched together using simple interrupted suture with polypropylene 3.0 USP thread. The skin graft area was then wrapped by framycetin sulfate (Sofra-Tulle^®^, Pantheon UK Limited, Swindon, UK for Sanofi-Aventis, Thailand). It was replaced on day 3, 6, 9, 12, and 18. Claneksi^®^ 62.5 mg/cat and flunixin meglumine 1 mg/kg BW were given 2 times daily for 8 days for maintenance [1]. On day 24^th^ post skin grafting, skin biopsy (punch biopsy) was conducted for histopathology examination and mouse anti-cytokeratin AE1-AE3 immunohistochemistry (Millipore, Germany).

### Histopathological examination

Skin biopsy samples were submerged in buffer neutral formalin 10% liquid for paraffin block histopathology sections. Tissue section that has been fixed on object glass was heated for 2 h in 60°C. Deparaffinization and rehydration were done using xylol I, xylol II, and xylol III, and ethanol absolute I, II, and III, ethanol 90%, ethanol 80%, and ethanol 70%. Morphological structure observation of donor skin by HE staining included: Formation of epithelial cell on epidermis and dermis layer, formation of hair follicle, neovascularization, and glands [12,13]. The formation of hair follicle, sebaceous glands, and sweat glands was observed under 200 times magnification with three replications of visual fields. Average value was counted and scored under the following category: (a) Few (0-2 of found hair follicle, neovascularization, sebaceous glands, and sweat glands); (b) moderate (2-4 of found hair follicle, neovascularization, sebaceous glands, and sweat glands); (c) many (more than 4 of found hair follicle, neovascularization, sebaceous glands, and sweat glands). MT staining was used to observe the density of connective tissue that shows vivid blue stain intensity (very dense density), blue stain intensity (dense density), and scant blue stain intensity (less dense density).

### Immunohistochemistry examination

Tissue sections that had been fixed to object glass were heated for 2 h in 60°C temperature. Xylol I, xylol II, and xylol III, and ethanol absolute I, II, and III, ethanol 90%, ethanol 80%, and ethanol 70% were used for deparaffinization and rehydration. Sections were washed by phosphate buffer solution (PBS), followed by endogenous peroxidase blocking with H_2_O_2_ 3% solution in aqua distillate for 30 min. Sections were then washed by PBS 3 times, 5 min each. Afterward, ultra V block was done for 5 min using goat serum and dripped by mouse anti-cytokeratin AE1/AE3 (Millipore, Germany), overnight in refrigerator. Section then washed by PBS. Secondary antibody used was biotinylated goat anti-polyvalent for 10 min, and then washed by PBS. Streptavidin peroxidase conjugate enzyme was added then PBS washed sections again. Substrate and 3,3’ diaminobenzidine and section was incubated for 10 min in room temperature in dark. Sections were afterward dehydrated by graded ethanol, cleared by xylol, then mounted permanently using Canada balsam [14]. Immunohistochemistry observation result was scored by the following category: Very high number of epithelial cell immunoreactive toward mouse anti-cytokeratin AE1/AE3 (deep brown color), high number of epithelial cell immunoreactive toward mouse anti-cytokeratin AE1/AE3 (brown color), and small number of epithelial cell immunoreactive toward mouse anti-cytokeratin AE1/AE3 (scant brown color).

### Statistical analysis

Quantitative data of histopathological changes and mouse anti-cytokeratin AE1/AE3 expression on donor skin tissue was analyzed descriptively.

## Results

### Donor skin morphology structure

**Figure-1:**
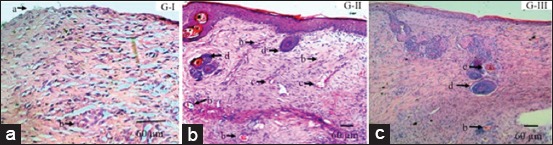
Morphological structure changes of donor skin post skin graft. Group I (G-I); skin epidermis layer epithelial cell was not well formed (a), neovascularization (b). Group II (G-II); neovascularization (b), hair follicle (c), and gland (d). Group III (G-III); neovascularization (b), hair follicle (c), and gland (d). Hematoxylin and eosin staining.

Group I donor skin showed inflammatory cell infiltration, few neovascularization, and few sebaceous glands, while hair follicle and sweat glands were absent. Group II showed inflammatory cell infiltration, a lot of neovascularization, low-density connective tissue, and numerous sweat gland and hair follicle growth can be found. Group III also showed inflammatory cell infiltration, moderate number of neovascularization, moderately dense connective tissue, and moderate number of sweat gland and hair follicle. Skin donor tissue composition observation showed epidermis layer (stratum corneum up to stratum basale), dermis layer (marked by the presence of hair follicle, sweat gland, sebaceous gland, and collagen fiber), and hypodermis layer (fat tissue, nerves, blood vessel, and collagen fibers were found and already underwent reorganization to form webbing). Epidermis layer epithelial cell in Group I had not formed completely whereas Groups II and III had. Morphological structure changes on donor skin post skin grafting are presented in Figure-1.

**Figure-2:**
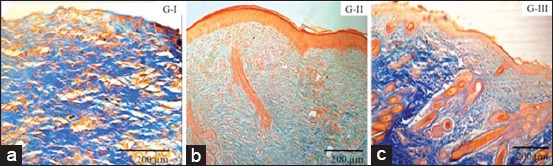
Density connective tissue of donor skin post skin graft. Group I (G-I): Very dense connective tissue formed, indicated by vivid blue staining. Group II (G-II): Lightly dense connective tissue formed, indicated by scant blue staining. Group III (G-III): Dense connective tissue formed, indicated by blue staining. Masson’s trichrome staining.

Microscope observation of donor skin tissue by MT staining showed different connective tissue density (intensity of blue staining) between each group. The result is presented in Figure-2. The highly dense connective tissue density (vivid blue intensity) found on Group I was because the surface of wound base was not good enough to receive donor skin. This caused some part of donor skin to undergo necrosis and be replaced by connective tissue. Granulation on Groups II and Group III wound base was good enough that donor skin could be received entirely. The density of Group I and Group III was lightly dense (scant blue staining) and dense (blue staining), respectively. The wound bases of the two groups were ready to receive donor skin, indicated by good granulation forming. Placement of donor skin on wound surface with good granulation speeds up neovascularization between donor skin and recipient base wound. Thus, epithelial cell necrosis of donor skin will lessen, resulting in decreased formation of connective tissue.

### Mouse anti-cytokeratin AE1/AE3 immunohisto chemistry

**Figure-3:**
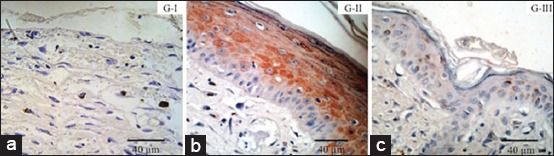
Cytokeratin AE1/AE3 expression of donor skin post skin graft. Group I (G-I); cytokeratin AE1/AE3 expression in dermis layer with scant brown intensity. Group II (G-II); cytokeratin AE1/AE3 in epidermis and dermis layer with very brown intensity. Group III (G-III); cytokeratin AE1/AE3 expression in epidermis and dermis layer with brown intensity. Mouse cytokeratin AE1/AE3 immunohistochemistry staining.

Cytokeratin AE1/AE3 expression was found more in Group II with very brown staining compared to Group III with brown staining and Group I with scant brown staining. Cytokeratin AE1/AE3 expression was found in epidermis layer epithelial cell (very brown stain) and scant brown on dermis layer of Group II. Cytokeratin AE1/AE3 expression generally found in membrane, epithelial cell intracytoplasm, and hair follicle as shown in Figure-3. Skin tissue of Group I showed that its epidermis layer had not formed completely so cytokeratin AE1/AE3 expression was only found in membrane and dermal layer epithelial cell intracytoplasm with scant brown intensity. Cytokeratin AE1/AE3 expression of Group III donor skin tissue was found in epidermis layer epithelial cell membrane (brown) and in dermis layer epithelial cell membrane (scant brown). Brown staining of cytokeratin AE1/AE3 expression showed that there were many epithelial cells immunoreactive against mouse anti-cytokeratin AE1/AE3.

## Discussion

This research is an experimental research to observe morphological structure, connective tissue density, and cytokeratin AE1/AE3 expression intensity post skin graft in Indonesia local cats with different donor skin placement period. Success of skin graft was identified histopathologically by findings of epidermis layer, hair follicle, sweat gland, sebaceous gland, and blood vessels in dermis layer [12]. Epidermis layer is the outermost layer of skin that synthesizes keratin on its stratum corneum layer. Clusters of dead cells form keratin; keratinous cell will always peel and then be replaced by continuously dividing cells in stratum basale. Between stratum corneum and stratum basale exist stratum spinosum and stratum granulosum. Epidermis cells also form keratin structure such as hair, feather, nail, beak, and horn [15]. Epidermis layer that is lost in necrosis will be replaced by squamosal cell. This cell originated from ectodermal which function to form keratin. Basal cells undergo proliferation, differentiation, and moved through all layer of epidermis. At the time basal cell proliferates, it develops into cell in epidermis layer, and keratinocyte also changes morphologically [16].

Skin consists of two layers: Epidermis layer and dermis layer. Dermis layer is the larger part, consisting 95% of skin, and the rest of 5% is epidermis layer. In dermis layer of the skin, there are sebaceous gland, sweat gland, and hair follicle. Loose skin attaches to hypodermis, which composed of loose connective tissue that connects skin with muscle. In normal condition, connective tissue is produced by fibroblast. Fibroblast has a role in extracellular matrix synthesis, deposition, and remodeling. After migrating to wound site, fibroblast will begin to synthesize extracellular matrix. Connective tissue is the main protein of extracellular matrix and has a big role in wound recovery [13,17].

During wound recovery, keratinocyte has a role in forming epidermis layer, cell migration, cell proliferation, and keratinocyte differentiation by the edge of wounds. The edge of the epidermis is composed of cells with the ability to migrate. Differentiation of new epidermis layer begins right before complex re-epithelialization happen. New epidermis layer formation during wound recovery is not without the help of growth factor, cytokine, extracellular protein matrix, and local materials that are secreted during inflammation by fibroblast, endothelial cell, and keratinocyte [5]. Different cytokeratin expression may be influenced by cell differentiation, cell growth stages, maturation, and type of epithelial cell. During keratinocyte migration to epithelial layer, epithelial cell undergoes maturation process that can be detected by observing the expression of cytokeratin [18].

Cytokeratin is an intermediate filament keratin protein found in epithelial cell cytoplasm. Keratinocyte is responsible in keratin formation, which is a characteristic of transitional mesenchymal-epithelial cell post-surgery. In the beginning, keratinocyte first migrates to the surface of wound epidermis layer. After new epidermis layer has been well formed, keratinocyte then proliferates to rebuild epidermis. The speed of proliferation in epidermis and dermis cell reaches its peak after re-epithelialization and matrix synthesis happen [8]. Approximately half of keratinocyte migrate from basal layer to other epidermis layer. Migration from basale layer is followed by sel structure change beginning from thinning, losing nucleus, and finally turning dry. When these cells reach the outermost layer, they are then known as the horn cell (stratum corneum). Second largest cell of epidermis layer is melanocyte that can be found in stratum basale [5].

First few days post skin graft surgery; donor skin must be bandaged to protect it from environment influence, letting optimal re-epithelialization process to occur [1]. Skin graft recovery begins from swelling that happens in first 48-72 h after surgery. This is caused by degradation of hemoglobin product. Afterward swelling will lessen as a sign that base wound circulation is established [1,16]. Skin transplantation is deemed unsuccessful if within 3-5 day’s post-surgery, donor skin looks ischemic or necrotic. Sometimes donor skin loses its epidermis layer for the 1^st^ week, which made it seems like failure happens during skin transplant. This could be confusing, however usually in general dermal graft will resurface, and donor skin will develop satisfactorily to cover impairment [1,19].

## Conclusion

Histopathological structure of donor skin post skin graft surgery showed satisfactory result. In Group II and Group III more neovascularization, inflammatory cell infiltration, formation of hair follicle, and formation of hair follicle, sweat gland, and sebaceous gland were found compared to Group I. MT staining showed connective tissue density (blue color) is very dense density in Group I, less dense density in Group II, and Group III with dense density. Cytokeratin AE1/AE3 expression was more often found in Groups II and III compared to Group I, especially in epidermis layer.

## Authors’ Contributions

Authors will declare the contribution of each author such as Erwin, Gunanti, and DN conceived and designed the anesthesia and surgery. Erwin, EW, and EH executed the experiment, analyzed and interpretation of data the tissue. All authors read and approved the final manuscript.
